# Parental stress during the COVID-19 pandemic: A one-year follow-up

**DOI:** 10.1371/journal.pone.0276190

**Published:** 2022-12-06

**Authors:** Ragnhild Bjørknes, Jens Christoffer Skogen, Ane Nærde, Gro Mjeldheim Sandal, Ellen Haug, Silje Mæland, Lars T. Fadnes, Stine Lehmann

**Affiliations:** 1 Department of Health Promotion and Development, Faculty of Psychology, The University of Bergen, Bergen, Norway; 2 The Norwegian Center for Child Behavioral Development, Oslo, Norway; 3 Department of Health Promotion, Norwegian Institute of Public Health, Bergen, Norway; 4 Alcohol & Drug Research Western Norway, Stavanger University Hospital, Stavanger, Norway; 5 Centre for Evaluation of Public Health Measures, Norwegian Institute of Public Health, Oslo, Norway; 6 Department of Psychosocial Science, Faculty of Psychology, The University of Bergen, Bergen, Norway; 7 NLA University College, Bergen, Norway; 8 Department of Global Public Health and Primary Care, Faculty of medicine, University of Bergen, Bergen, Norway; 9 Research Unit for General Practice in Bergen, The Norwegian Research Centre, NORCE, Bergen, Norway; 10 Bergen Addiction Research, Department of Addiction Medicine, Haukeland University Hospital, Bergen, Norway; King Fahd University of Petroleum & Minerals, SAUDI ARABIA

## Abstract

**Objective:**

This two-wave longitudinal study aimed at increasing knowledge about levels of parental stressors and rewards among mothers and fathers of children aged 1–18 during the first year of the COVID-19 pandemic in Norway.

**Background:**

The COVID-19 pandemic and infection-control measures have caused changes to family life. Managing homeschooling or caring for younger children while working from home may have posed significant strain on parental stress, negatively impacting the quality of parent-child relationships and parents’ sensitivity to their children’s needs.

**Method:**

We employed data collected in April 2020 and April 2021 from the longitudinal population-based survey in Bergen/Norway (Bergen in ChangE-study). 7424 parents participated (58.6% mothers and 41.5% fathers).

**Results:**

The overall levels of parental stressors and rewards did not change significantly. Over the two time points, the factors associated with decreased parental stressors were being male, aged 40–49 years, having a relatively high income, and reporting initial difficulties with closed kindergartens or schools. For parents aged 18–29 years, the level of parental stressors increased.

**Conclusion:**

The study suggests that the overall levels of parental stress remained unchanged during the first year of the pandemic. Even so, the study also uncovered that younger parents represented a vulnerable subgroup.

**Implications:**

To prevent detrimental consequences in the wake of the pandemic, it could be important to increase awareness and competence among professional staff in kindergartens, primary schools, and child health clinics targeting young parents and their children.

## Introduction

The COVID-19 pandemic and subsequent disease-control measures have impacted the health and wellbeing of children and parents worldwide. Parents have reported challenges related to their financial situation, social isolation, combining working from home with homeschooling, and stress symptoms, including anxiety, worrying, depression, and poor sleep [1–3]. These challenges are likely to affect family life [4], yet there is limited knowledge about the extent to which the pandemic and subsequent disease-suppressive measures are associated with family functioning over the course of the pandemic.

Parenting stress can be conceptualized as a negative psychological response to the typical obligations associated with raising children and represents a normal reaction to the demands of parenting [5–7]. Central to most definitions of parenting stress is the perceived balance between the practical and emotional requirements of parenting and the resources available for meeting them [7]. When the demands exceed the resources, as could likely be the case during a dramatic incident like the worldwide COVID-19 pandemic involving increased caregiving burden and major changes in daily routines, parent’s typically experience high levels of stress, including low levels of parental rewards. Emprical evidence indicate that parenting stress is significantly associated with the well-being and adjustment of both parents and children, including adult functioning, the quality of parent-childrelationships, and child behavior and development [e.g., 5–11]. The current study uses data from two time points to investigate levels of parental stress (stressors and rewards) among mothers and fathers in Norway during the first year of the Covid-19 pandemic (from April 2020 to April 2021).

### COVID-19 and its effect on parenting stress

A conceptual framework of COVID-19 disruption and resilience [4] drawing from pertinent literature relating to acute crisis and long-term, cumulative risk was introduced by Prime et al. to present a model of how social disruption due to COVID-19 may impact child adjustment. This model [4] describes various processes of risk and resilience in family wellbeing during the pandemic, focusing among other things on the importance of parenting stress for caregiver well-being and ultimately childrens adjustment. This framework postulates that financial insecurity, increased caregiving burden, and confinement-related stress can affect caregiver’s wellbeing, including their level of parental stress [4, 12]. This might negatively impact the quality of parent-child relationships and parents’ sensitivity to their children’s needs which could indirectly affect children’s socio-emotional functioning [4, 6, 13]. Indeed, studies from the earliest phase of the COVID-19 pandemic suggest that the demands on caregivers were particularly challenging for many parents during this stressful and uncertain period [4, 14, 15].

This is not surprising given that families were collectively experiencing a new range of stressors that threatened safety, economic well-being, health and every-day life due to the COVID-19 pandemic. In particular, research conducted during spring/summer 2020 in Italy and Germany showed that parents experienced more parenting-related exhaustion [14–16], with mothers being more severely affected than fathers [15]. Exhaustion was related to social distancing measures to hinder the spread of the virus, including the closure of school facilities [16]. Findings from Italy show that known risk factors for parental stress were associated with a high parental stress level during COVID-19 [15]. These factors included being single, having younger children, having a child with special needs, and having many children.

Emerging evidence from several other European countries suggests that homeschooling during the first month of the pandemic was related to an increase in negative parent-child interactions [3]. Similar results were found during the first lockdown in Norway in March and April 2020, when about half of the adult population worked from home, and all kindergartens and schools were closed [17]. During this period, parental stress was associated with being a mother, living with more than one child per parent, having children with special needs, and parents’ pre-existing mental disorders [17]. A follow-up study in June 2020 showed that the initially high levels of stress among Norwegian parents decreased along with less suppressive measures [18].

The present study examines how this prolonged pandemic situation is linked with stability or change in levels of parental stress (stressors and rewards) across the first year of the COVID-19. Based on the findings from studies examining parental stress during the initial phase of the COVID-19, we examine if parental gender, age, education, and family income, as well as COVID-19 related social distancing (working from home or difficulties with closed kindergartens/schools) and economic strain (being temporarily laid off), interact with change in levels of parental stress.

### The COVID-19 situation in Norway at the time of the present study

National measures to suppress the spread of the COVID-19 infection were implemented in Norway on the 12th of March 2020, followed by local suppressive measures during the winter of 2020 and spring of 2021 [19]. In March and April 2020, about half of the adult population worked from home, and all kindergartens and schools were closed. In spring 2021, there was a slow albeit limited return to physical presence at workplaces, kindergartens, and schools as the vaccination initiative commenced. But due to recurrent regional outbreaks, many families in the municipality of Bergen (where this study is conducted) continued to live under unpredictable and severe social restrictions, including periods with closed schools and kindergartens, yielding significant disruptions in everyday life.

On a national level, the pandemic contributed to an increased referral rate to child mental health care [20], rising unemployment rates [21], and increased burdens on parents regarding childcare, which have affected mothers the most [22]. Moreover, research carried out in Bergen shows that both youths [23–25] and adults [2] have struggled to adjust to the new situation during the first months of the pandemic, and that changes in the daily routines generally have caused stress and worries. However, there is a lack of empirical knowledge about how the pandemic situation is linked with parental stress over an extended period of the pandemic.

### Aims

This two-wave longitudinal study examines parental stress among mothers and fathers with children aged 1–18 years in the municipality of Bergen, Norway during the first year of the pandemic (from April 2020 to April 2021). We examine (1) the stability and change in levels of parental stressors and rewards across this period, (2) whether the parent’s gender, age, education, and family income are associated with changes in levels of parental stressors and rewards, and (3) whether the COVID-19 infection-control measures and their consequences (i.e., being temporarily laid off, working from home, or difficulties with closed kindergartens/schools) are associated with changes in levels of parental stressors and rewards.

## Methods

### Procedure

Data were taken from a population-based survey in Bergen/Norway (Bergen in ChangE-study) [2]. In April 2020, altogether 81,170 individuals were randomly selected from a total of 224,000 adult inhabitants in Bergen, Norway, and invited to participate in an online survey assessing the consequences of the COVID-19 outbreak (time point 1) in the general population. Eligible participants were drawn from a contact register through the National Population Registry of Norway. Bergen is the second largest city in Norway and is situated on the west coast. The first data collection started on the 27^th^ of April 2020 in the 7^th^ week of the national lockdown and ended on the 11^th^ of May 2020. At that time, national restrictions included social distancing, closed educational, cultural, and training/sport/gym facilities, requirements to work from home, and the first introduction of quarantine requirements. Of the 29,535 (36%) individuals who participated, 7,424 reported having children aged 1–18 years in their household and were thus eligible for this sub-study addressing parental stress.

A second data collection was conducted between the 21^st^ of April and the 6^th^ of May 2021 (time point 2). At that time, local restrictions included social distancing, closed training/sport/gym facilities, requirements to work from home if possible, and quarantine requirements in cases of virus exposure. This implied periods with homeschooling and home-based childcare for younger children in many families in Bergen. Hence, at follow-up, the temporarily ease of restrictions during summer 2021 had been replaced by more invasive disease-control measures due to a new rise in infection numbers.

### Measures

#### The parental stress scale

The Parental Stress Scale (PSS) [13] measures stressful and unsatisfying experiences (parental stressors) and positive and rewarding aspects of being a parent (parental rewards), and focuses on parent’s perceptions of their parental role rather than the *sources* of stress. The scale is brief and easy to administer, and is freely available. It is much used within both research and clinical practice. Parents are asked to rate 18 items about their relationship with their child on a Likert scale ranging from 1 ("strongly disagree") to 5 ("strongly agree"). The subscale parental stressors, includes ten items addressing negative and stressful aspects of parenting, such as “*I feel overwhelmed by the responsibility of being a parent*” and “*The major source of stress in my life is my child(ren)*”. The subscale Parental rewards comprises eight items, such as “*My child(ren) is (are) an important source of affection for me*”, and “*I enjoy spending time with my child(ren)”*. Based on previous research testing the psychometric properties of the Norwegian version of the PSS [26] and in line with other research [27, 28], the scale was used as a two-dimensional measure of parental stressors and parental rewards (i.e., lack of rewards when reversing the positively worded items or rewards when not reversing). At time point 1, Cronbach’s alpha was 0.82 for parental stressors and 0.72 for parental rewards, while the corresponding values at time point 2 was 0.83 and 0.72, respectively.

The sum score for the parental stressors sub-scale ranges from 10 to 50, where a high score signifies a high perceived level of stress associated with caring for children. The sum score for the parental rewards sub-scale ranges from 8 to 40, where a high score reflects a high level of perceived rewards associated with parenthood. The scale was translated into Norwegian by the third author (AN), and the back-translated version was approved by Judy Berry, one of the authors of the original PSS.

#### Covariates measured at time point 1

The parents’ gender, age, educational attainment, and family income were measured by self-report. Working from home, temporarily being laid off, and finding kindergarten/school closure difficult to handle were also measured by self-report. The wording of the questions were; which of these control measures has been difficult to handle?

#### Ethics

The Regional Committee for Medical and Health Research Ethics, Western Norway, approved the study (project number 131560). Responders consented to participate by providing their written informed consent. This was done by ticking off at a consent form at the start of the survey.

### Statistical analyses

First, time point 1 descriptive statistics of the included study variables were calculated. Categorical variables are presented using the number of participants and percentages across levels, while continuous variables are presented using means and standard deviations (SDs). Next, mixed linear models were estimated with the two dimensions of the PSS (parental stressors and parental rewards) as the dependent variables. Mixed linear modeling was chosen as the preferred statistical approach in order to use all valid observations at both time point 1 and follow-up under the assumption of missing at random [29].

In the mixed linear models, time was the primary independent variable, and crude and adjusted models were estimated. Separate models adjusted for covariates (parental gender, age, educational attainment, family income, and COVID-19-related variables), as well as a fully adjusted model, were estimated. Separate mixed linear models investigating the potential interaction between time and covariates were estimated as well (e.g. parental stress as outcome and time × gender as interaction term). In the case of a significant interaction term (time × covariate using alpha = 0.05), estimated scores on parental stressors, parental rewards, and contrasts of marginal predictions were estimated for each level of the covariate (separately for each covariate). Contrasts are presented as raw scores and standardized Z-scored scores. Missing information on variables ranged from n = 9 (0.1%) to n = 248 (3.8%). To retain the maximum number of observations, pairwise deletion was employed for all analyses. All analyses were done using Stata 16 [30].

### Participants

Demographic information for the participants is presented in Table 1. A total of 7424 parents participated in the first data collection during April to May, 2020. Of these, 58% were mothers and 41% were fathers, 7.6% reported being temporarily laid off at time point 1, while 68% worked from home. Further, 46% experienced that it was difficult to handle closed kindergartens/schools and 9.6% of the participants had immigrated to Norway. The parents had between 1 to 6 children with a mean of 1.8 (*SD* 0.8). At time point 2 (April 2021), a total of 4,656 (63% retention rate) parents agreed to participate. Compared to the parents only participating at time point 1, the parents participating at both time points were more likely to be mothers, older, report higher educational attainment and higher family income (all *p*-values <0.001). The differences were, however, moderate where the proportion of mothers were 60% for both time points compared to 56% among the parents participating only at the first time point. For age categories, educational attainment and family income in quintiles, there was a 56% probability that a random draw from those participating at both time points had a higher rank order (e.g. came from a higher age group) compared to those participating only at time point 1.

**Table 1:** Sample characteristics and parental stress scale (PSS) scores at time point 1.

	N	Percent
Gender		
Female	4347	58.6
Male	3077	41.5
Age categories		
18–29 years	273	3.7
30–39 years	2420	32.6
40–49 years	3283	44.2
50–59 years	1308	17.6
60+ years	140	1.9
Educational attainment		
Primary school	240	3.2
Junior high school	1424	19.2
College/university <4 years	1825	24.6
University ≥4 years	3926	53.0
Family income in quintiles		
0-19th	1696	23.6
20-39th	1303	18.1
40-59th	1356	18.8
60-79th	1494	20.8
80-100th	1352	18.8
Temporarily laid off		
No	6857	92.4
Yes	567	7.6
Working from home		
No	2387	32.2
Yes	5037	67.9
Difficulty with closed kindergarten/school		
No	4020	54.2
Yes	3404	45.9
	Mean	Standard deviation
PSS scores at time point 1		
Parental stressors (N = 7196)	22.5	7.1
Parental rewards (N = 7215)	38.4	2.6

## Results

### Stability and change in levels of parental stress

At time point 1, the mean scores were 22.5 (*SD* 7.1) for parental stressors and 38.4 (*SD* 2.6) for parental rewards. There was no overall change in the levels of parental stressors or rewards between time point 1 and time point 2 (Table 2). Neither separate adjustments for covariates nor adjustment for all covariates changed the estimates, and estimates remained non-significant across models.

**Table 2:** Change in the levels of parental stressors or rewards between time point 1 and time point 2.

	Coefficient	*p*
One-year time trend		
Parental stress	-0.07	0.337
Parental rewards	0.02	0.562
Time trend adjusted for gender		
Parental stress	-0.07	0.339
Parental rewards	0.01	0.677
Time trend adjusted for age		
Parental stress	-0.05	0.548
Parental rewards	0.02	0.525
Time trend adjusted for education		
Parental stress	-0.08	0.323
Parental rewards	0.02	0.573
Time trend adjusted for income		
Parental stress	-0.06	0.420
Parental rewards	0.01	0.680
Time trend adjusted for temporarily laid off		
Parental stress	-0.07	0.342
Parental rewards	0.02	0.567
Time trend adjusted for working from home		
Parental stress	-0.08	0.318
Parental rewards	0.02	0.568
Time trend adjusted for difficult closed kindergarten/school		
Parental stress	-0.07	0.347
Parental rewards	0.02	0.570
Time trend (fully adjusted)		
Parental stress	-0.04	0.603
Parental rewards	0.01	0.791

Note: Results from mixed linear models with parental stress scale as outcome and time as the primary independent variable and adjusting for other covariates separately or as a fully adjusted model.

### Parental background and COVID-19 control measures associated with changes in levels of parental stress

For parental stress, significant interactions were found between time and characteristics of the parent, including gender, parental age, family income, working from home, being temporarily laid off, and experiencing closed kindergartens or schools as difficult to handle (Table 3).

**Table 3:** Interaction of parent’s gender, age, education, family income and COVID-19 control measures on parental stressors and rewards. Each interaction term entered into separate analyses.

	Parental Stress		Parental Rewards	
	Main effect	Interaction term	Main effect	Interaction term
Gender	0.26	-0.63	-0.67	0.10
	(p = 0.7327)	**(p<0.0001)**	**(p<0.0001)**	(p = 0.1255)
Age				
18–29	ref	ref	ref	ref
20–39	1.14	-2.18	0.08	0.01
40–49	-0.95	-2.52	-0.03	0.05
50–59	-2.87	-2.05	-0.14	0.04
60+	-2.18	-2.34	-0.40	0.12
	**(p<0.0001)**	**(p<0.0001)**	(p = 0.0862)	(p = 0.9694)
Education				
Primary school	ref	ref	ref	ref
Junior high school	-0.21	0.05	0.18	0.21
College/university <4 years	0.30	-0.03	0.20	0.06
College/university ≥4 years	0.29	-0.23	0.12	0.18
	(p = 0.2795)	(p = 0.5041)	(p = 0.4457)	(p = 0.3376)
Income				
0-19^th^	ref	ref	ref	ref
29-39^th^	-0.50	-0.09	0.23	0.11
40-59^th^	-0.61	-0.07	0.14	0.07
60-79^th^	-1.29	-0.40	0.21	0.13
80-100^th^	-2.05	-0.69	0.18	0.27
	**(p<0.0001)**	**(p = 0.0239)**	**(p = 0.0025)**	(p = 0.1351)
Temporarily laid off	0.89	-0.67	-0.11	0.02
	(p = 0.0737)	**(p = 0.0215)**	(p = 0.3275)	(p = 0.9036)
Working from home	0.75	-0.74	0.03	0.01
	**(p = 0.0326)**	**(p<0.0001)**	(p = 0.6028)	(p = 0.8769)
Difficult closed kindergarten/school	4.48	-0.75	-0.44	0.21
	**(p<0.0001)**	**(p<0.0001)**	**(p<0.0001)**	**(p = 0.0015)**

Results from mixed linear models with parental stress scale as outcome and time as the primary independent variable. P-values from contrasts of marginal linear predictions in parentheses. P-value expresses joint effects for ordinal level variables (age, education and income). Bold indicates p-values <0.05.

**Fig 1:**
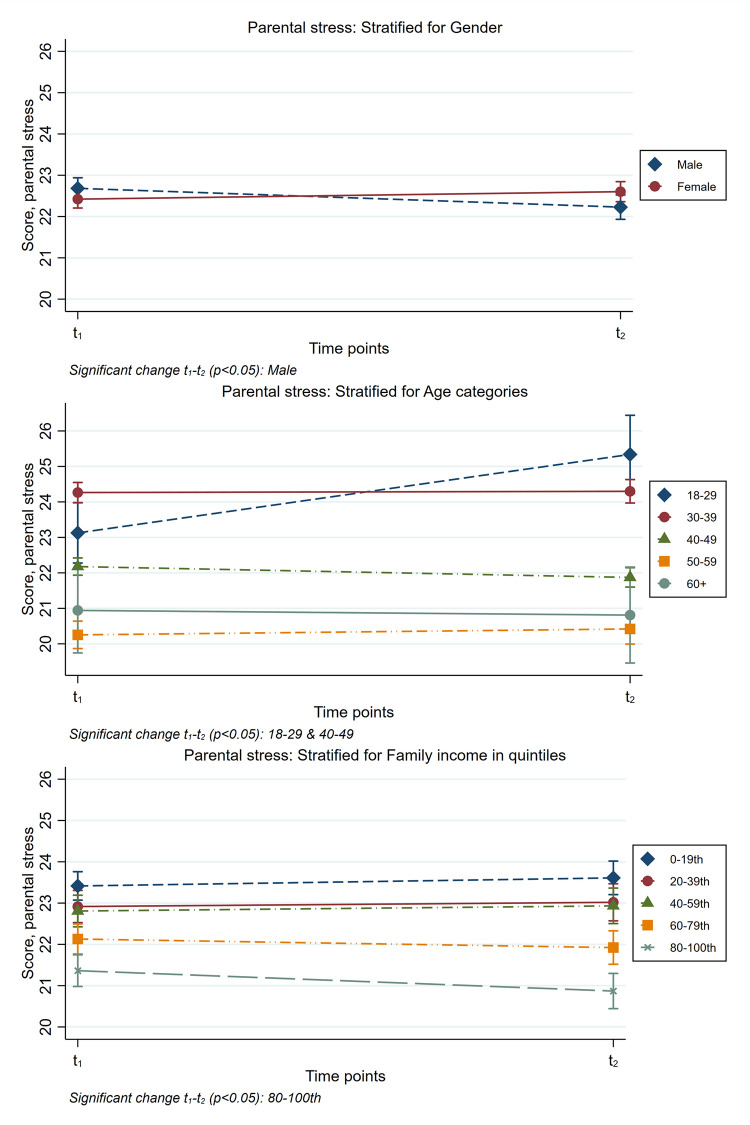
Stratified results for parental stressors. Time point 1 in April-May 2020. Time point 2 in April-May 2021.

**Fig 2:**
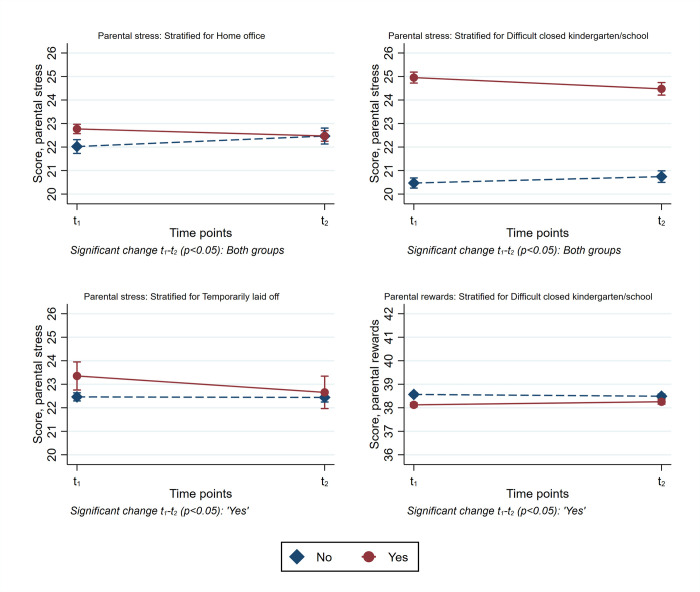
Stratified results for parental stressors and parental rewards. Time point 1 in April-May 2020. Time point 2 in April-May 2021.

For parental rewards, only one significant interaction of time and closed kindergartens/schools was identified. These interactions were retained for further stratified analyses. The stratified results for parental stressors and parental rewards are presented in Table 4 and in Figs 1 and 2, respectively.

**Table 4:** Stratified results for parental stressors and parental rewards. Contrasts of marginal linear predictions: time point 2 compared to time point 1. Separate stratification for each covariate.

	Contrasts	Standardized contrasts^a^	P-value
Parental stressors			
Gender			
Male	-0.455	-0.064	**<0.001**
Female	0.180	0.025	0.0670
Age			
18–29 years	2.212	0.310	**<0.001**
30–39 years	0.035	0.005	0.802
40–49 years	-0.305	-0.043	**0.006**
50–59 years	0.167	0.023	0.340
60+ years	-0.131	-0.018	0.816
Income, quintiles			
0-19th	0.195	0.027	0.250
20-39th	0.102	0.014	0.581
40-59th	0.125	0.018	0.475
60-79th	-0.207	-0.029	0.206
80-100th	-0.494	-0.069	**0.004**
Temporarily laid off			
No	-0.023	-0.003	0.769
Yes	-0.697	-0.098	**0.014**
Working from home			
No	0.446	0.063	**0.001**
Yes	-0.298	-0.042	**0.001**
Difficult closed kindergarten/school			
No	0.272	0.038	**0.008**
Yes	-0.479	-0.067	**<0.001**
Parental rewards			
Difficult closed kindergarten/school			
No	-0.077	-0.029	0.084
Yes	0.131	0.050	**0.006**

Note: Results from mixed linear models with parental stress scale as outcome and time as the primary independent variable. Contrasts of marginal linear predictions, time point 2 compared to time point 1. Parental stressors scores stratified separately for gender, age, income, temporarily laid off, working from home, and difficulty with closed kindergarten/school. Parental rewards stratified separately for difficult with closed kindergarten/school.

^a^Z-scored values based on scores at time point 1.

Males, parents aged 40–49 years, and those with the highest quintile of family income reported a decrease in levels of parental stressors from time point 1 to 2 (Table 4 and Fig 1). Furthermore, there was a decrease in the level of parental stressors for parents who reported having been temporarily laid off, working from home, and finding it hard to handle closed kindergartens/schools difficult at time point 1 (Table 4 and Fig 2). Conversely, an increase in parental stressors from time point 1 to time point 2 was observed for those aged 18–29 years, parents who were *not* temporarily laid off from work, and those who did *not* report that closed kindergartens/schools was difficult at time point 1 (Table 4 and Fig 2). For parental rewards, an increased score was observed for those who reported closed kindergartens/schools as being difficult to handle at time point 1 (Table 4 and Fig 2). Looking at the standardized contrasts in Table 4, the actual changes in scores were very small for all subgroups with the exception of parents aged 18–29 years, where the increase in levels of parental stressors was small to medium (i.e., about 0.3 SD).

## Discussion

This two-wave longitudinal study examined stability and change in parental stress (i.e., levels of stressors and rewards) among parents with children aged 1–18 years during the first year of the COVID-19 pandemic in Norway. Our results show that overall, neither the level of parental stressors nor parental rewards changed notably in this time period. However, being a father, being aged 40–49 years, having a relatively high income, or reporting initial difficulties with closed kindergartens or schools, were associated with decreased levels of parental stressors from April 2020 to April 2021. While the changes were small for all subgroups, the youngest parents (those aged 18–29 years) reported the highest increase in stress level during the first year of the pandemic.

Studies from several European countries have reported parenting-related exhaustion during the first lockdown in the spring and summer of 2020 [14, 15, 31]. In Norway, this was followed by a significant decrease in parental stress in the summer [18]. Our main result indicates overall stability in parental stress among Norwegian parents across the first year of the pandemic. Differences in national findings might reflect variations in the strictness and duration of the imposed measures or other contextual factors (e.g., available support for families). It is possible that the modest changes found in previous studies [17, 31] and the present research reflect a “normalization” of family life following the first lockdown as people adapted.

In their study in Norway, Johnson et al. [18] argue that the results showing a decrease in levels of parental stress from March to June 2020 indicate that the overall stress levels declined when the physical distancing measures were phased out. It could also be that parents use various coping strategies and mostly manage to adapt to the living conditions and restrictions imposed by national and local control measures. This interpretation is consistent with the framework of COVID-19 disruption and resilience by Prime et al, which posits that most families will do their best to mitigate the amount and type of disruption [4]. That being said, we need to point out that the overall level of parental stressors in this sample of Norwegian parents was low at both time points, and the level of parental rewards was consistently high.

Nonetheless, on a subgroup level, parents and families seem to have been somewhat differently affected by the pandemic and the restriction measures. Parents with certain socio-economic characteristics were more or less likely to experiencing stress in this phase. A decrease in parental stressors level was found for those reporting having the highest quintile of family income and parents who had been temporarily laid off in April 2020. We do not know whether the parents who were temporarily laid off during the first phase of the pandemic had in fact regained their job or were employed elsewhere one year later. However, we can assume that some of those who were temporarily laid off during the first phase were positively impacted by the national measures implemented to buffer economic instability.

The same trend was found for parents working from home during the first week of the pandemic. It is possible that working from home, combined with having challenges related to childcare and homeschooling in the initial phase of the pandemic, made it difficult for parents to adjust family life and maintain work/family balance. As our results show, however, most parents seem to have coped well during the first year after the onset of the pandemic. In spring 2021, there was also a slow return to physical presence at workplaces, kindergartens, and schools, that can have reduced the stressors, however, recurrent regional outbreaks continued with unpredictable and severe social restrictions, including periods with closed schools and kindergartens.

Overall, our results are in line with the model by Prime and colleagues on COVID-19 disruption and resilience [4] and support that most families seem to adjust well to the new normality.

This does not seem to hold for all parents, however, and those aged 18–29 years, who most likely have pre-school children, reported increased levels of parental stress. This group of parents could also be characterized by generally having lower family income, and a disproportionally large portion may be students or have less secure jobs. Also, it is possible that being younger and thus less experienced as a parent and caregiver, as well as the increased burden of caring for younger children, have made these families more vulnerable to experiencing parental stress. Nevertheless, these findings are important as increased parental stress during the pandemic has been related to decreases in wellbeing and increased problem behavior among children [32]. This implies that younger parents experiencing heightened stress during the pandemic might need timely follow-up to prevent enduring impacts on child and family wellbeing.

Our findings do not confirm that there is a disproportionate caretaking burden for mothers, but fathers reported a decrease in parental stressors during the first year of the pandemic, which confirms the gender difference found in previous studies. A multicountry study shows that women generally have spent more time than men on household chores and childcare during the COVID-19 pandemic and have also reported lower levels of happiness [33]. Likewise, studies from Italy and Norway have found that maternal stress increased during lockdown [17, 34]. Thus, the impact of home confinement may have placed a higher burden on mothers, possibly because they are more often the primary caregiver [17, 18, 33, 34].

Parents who found it difficult that kindergartens/schools were closed during the first weeks of the pandemic had a higher stress score at time point 1 than those who did not. While the score decreased across time, it was still higher at time point 2 than for parents who did not report the same type of difficulties. In fact, the parents who did not have difficulties related to the closing of kindergartens and schools reported that the level of parental rewards increased during the year. Many families experienced that kindergartens and schools closed, reopened, and closed again in an unpredictable pattern. In general, lack of predictability is closely linked with the experience of stress [35]. Moreover, kindergartens and schools are seen as significant recovery environments for children during disaster preparation and recovery [36]. More knowledge is needed regarding factors associated with parents’ experiences with homeschooling during the pandemic. For example, perceived social support, parents’ educational levels, child age, special needs, and homeschool communication may all explain variations in how parents experienced repeated closures of kindergartens and schools.

### Strengths and limitations

A notable strength of the present study is the longitudinal design. Also, the sample is extensive and includes a balanced number of mothers and fathers. Still, we did not have assessments of parental stress before the outbreak of the pandemic. Adding to this, there is no normative data on the PSS from Norway. Because of this, it is not possible to ascribe changes in parental stress levels specifically to the effects of COVID-19 or disease-control measures. In addition, we do not know whether the stress at T1 represents an increase from pre-pandemic levels, and the stability across time suggests that high stress was maintained. Although we would have liked to assess more interactions, the study size does to limited degree allow for assessment of complex interaction effects.

Similarly, the first measure of parental stress and potential predictors were assessed simultaneously, so we cannot make conclusions about the direction of those associations. In addition, we only have assessments at two time points, and it could be that there were significant fluctuations in parental stressors or rewards throughout the study period. Parent reports at T1 on rewards were essentially at the ceiling level of the measure (mean score of 38.4 out of a maximum of 40.0). Thus, parental rewards had almost no room to increase, which could be a possible outcome at T2 if pandemic restrictions eased.

Lastly, we were not able to pinpoint the age of the child(ren) that parents were thinking about when completing the survey. It is challenging to assess parents’ experiences (positive or negative) with parenting without having information about the age of their child(ren), and this should be taken into consideration when interpreting the results from the current study. Nevertheless, the finding for parents aged 18–29 years who reported increased levels of parental stress should be noted. These parents most likely have preschool children and are also generally less experienced as parents.

When considering generalizing our findings, one should be aware that the sample was recruited from a relatively small and demographically restricted area, even if it is the second largest city in Norway. The sample was somewhat biased towards parents with higher educational levels and household incomes when compared to the non-consenting parents. In addition, the severity of regionally imposed restrictions could differ widely based on infection rates in the geographic area. Thus, the results will most likely not be representative of all families in Norway. Furthermore, additional aspects of family life should be considered, including the availability of social support and the contribution of child-related factors. Future research should also consider how parental stress influences parenting practices during a pandemic.

## Conclusion

This study is a novel contribution in implying that even though families have been experiencing a worldwide crisis, the overall level of parental stressors and reward was unchanged when measured in April 2020 and again in April 2021 in Norway. However, health and social service providers should be aware of parents who might have heightened stress vulnerability. In the present study, these included the younger parents. It may be highly important to increase resources and competency in well-baby clinics targeting vulnerable young parents and their children in the wake of the pandemic to prevent detrimental consequences for children and families’ wellbeing. Furthermore, kindergarten and primary-school teachers are on the front line for reaching out to parents who are struggling with the economic and social consequences of the imposed preventive measures. Therefore, these practitioners should be trained for early detection and support to families who still struggle with pandemic-related problems.
